# Differences in digit ratios between gay men who prefer receptive versus insertive sex roles indicate a role for prenatal androgen

**DOI:** 10.1038/s41598-021-87338-0

**Published:** 2021-04-14

**Authors:** Ashlyn Swift-Gallant, Victor Di Rita, Christina A. Major, Christopher J. Breedlove, Cynthia L. Jordan, S. Marc Breedlove

**Affiliations:** 1 Department of Psychology, Memorial University of Newfoundland, 232 Elizabeth Ave., St. John’s, NL A1B 3X9 Canada; 2 Psychology Department and Neuroscience Program, Michigan State University, 293 Farm Lane, East Lansing, MI 48824 USA

**Keywords:** Neuroscience, Sexual behaviour, Psychology, Human behaviour

## Abstract

Among non-human mammals, exposure to androgens during critical periods of development leads to gynephilia (attraction to females), whereas the absence or low levels of prenatal androgens leads to androphilia (attraction to males). However, in humans, retrospective markers of prenatal androgens have only been associated with gynephilia among women, but not with androphilia among men. Here, we asked whether an indirect indication of prenatal androgen exposure, 2D:4D, differs between subsets of gay men delineated by anal sex role (ASR). ASR was used as a proxy for subgroups because ASR groups tend to differ in other measures affected by brain sexual differentiation, such as gender conformity. First, we replicated the finding that gay men with a receptive ASR preference (bottoms) report greater gender nonconformity (GNC) compared to gay men with an insertive ASR preference (tops). We then found that Tops have a lower (male-typical) average right-hand digit ratio than Bottoms, and that among all gay men the right-hand 2D:4D correlated with GNC, indicating that a higher (female-typical) 2D:4D is associated with increased GNC. Differences were found between non-exclusive and exclusive same-sex attraction and GNC, and ASR group differences on digit ratios do not reach significance when all non-heterosexual men are included in the analyses, suggesting greater heterogeneity in the development of non-exclusive same-sex sexual orientations. Overall, results support a role for prenatal androgens, as approximated by digit ratios, in influencing the sexual orientation and GNC of a subset of gay men.

## Introduction

One of the largest sex differences in human behavior is sexual orientation—on average males tend to be attracted to females whereas females tend to be attracted to males, with only 2–10% of the population same-sex oriented^1^. One theory of sexual orientation is the neuroendocrine hypothesis^2^, based on the findings that among mammalian species studied in the lab, androgens in early development organize masculine sexual preferences in adulthood. To test whether prenatal androgens are associated with human sexual orientation, an approximate retrospective measure of prenatal androgen, the ratio of the length of the index finger (2D) divided by the length of the ring finger (4D), has been measured among men and women with a same-sex sexual orientation and compared to heterosexual counterparts. The literature has been consistent for female sexual orientation—lesbians have a lower (more male-typical) 2D:4D than heterosexual women in numerous studies and two meta-analyses^3^. A role for prenatal androgens may be particularly pertinent for gender nonconforming lesbians—self-identified “butch” lesbians have a lower digit ratio than self-identified “femme” lesbians. In contrast, the data do *not* support a role for prenatal androgens in the development of differences in sexual orientation among men, as concluded by the same two meta-analyses^4,5^.

Given the association with 2D:4D and gender roles among female sexual orientation, we hypothesized that a subset of gay men who are more gender nonconforming may show a larger, less male-typical, 2D:4D than more gender conforming gay men. A growing body of work indicates that anal sex role (ASR) preferences may serve as a proxy for subgroups of gay men who differ in development and gender conformity^6–9^. Thus, in the present study we asked whether gay men with different ASR preferences may differ in 2D:4D. We hypothesized that gay men with a Bottom ASR (receptive), who tend to be more gender nonconforming (GNC), would have a higher (more female-typical) average digit ratio than ASR Tops (insertive), who tend to be more gender conforming. We predicted that gay men with a Versatile ASR preference (i.e., preference for insertive and receptive) would be intermediate between these two groups in both GNC and 2D:4D.

## Methods

### Participants

Participants were recruited via local public events in Lansing MI USA (Pride 2017), Charlotte MI USA (Frontier Days 2017), Chicago IL USA (Pride Fest 2017), Palm Spring CA USA (Pride 2018) and Toronto ON Canada (Pride 2017). Researchers had a booth at each event and as attendees passed by, they were asked if they would be interested in participating in our study. Prospective participants were informed they would be asked to fill out an anonymous survey asking about their sexual preferences, gender traits and other demographic information, as well as be requested to provide a saliva sample and photocopy of their hands, and offered a lottery scratcher ticket as a reward. We recruited a total of 694 participants, 407 of which completed the focal measures in this study (i.e., GNC and digit ratios); of this total, 259 were male and 149 self-identified as gay men [i.e., (1) indicated that they were assigned male at birth, (2) identify with male gender, (3) identify as gay and 4) report an exclusive attraction to males in the last year], 125 of which indicated their ASR preferences, and 122 indicated their ASR behavior. Of the 125 participants included in this study (i.e., gay men who indicated their ASR preference), ages ranged from 19 to 71 (*M* = 39.3; *SD* = 14.1), and the ethnicity of participants was primarily Caucasian: 96 Caucasian, 2 Black, 6 Asian, 1 Aboriginal, 10 Latin American, 2 Arab, and 9 did not respond. Informed consent was obtained from all participants and all procedures were approved by and followed the guidelines and regulations set out by the Michigan State University ethics review board.

### ASR preference and behavior

On the survey, participants were asked about their ASR preferences with the following question “*If you engage in and/or fantasize about anal sex, which role do you prefer (or fantasize about)*?” Options provided included *Bottom, Versatile, Top* and *I decline to answer*. Next, participants were asked about which ASR they typically engage in (i.e., ASR behavior): “*If you engage in anal sex, which role do you usually take*?” Options provided included *Bottom, Versatile, Top, Not sufficient experience with anal sex to answer* and *I decline to answer*.

### Digit ratios

Photocopies were taken of participants’ hands with a HP DeskJet 3630 All-in-One tabletop photocopier. Specifically, participants were asked to place their hands on a plexiglass sheet which had a standard ruler and pegs to place between the index and middle finger. They were asked to align their middle finger with a thick black line running down the midline on both the right and left side of the sheet, and then to keep their fingers close together while photocopied. Experimenters later measuring digit lengths from the photocopies were blind to participant characteristics.

### Measures of gender nonconformity

The Recalled Childhood Gender Identity scale^10^ was used as a retrospective measure of childhood gender nonconformity; we used the 18-items corresponding to Factor 1 in^10^. For a measure of perceived adulthood gender conformity, we asked participants “*How masculine or feminine do you consider yourself to be mentally, compared to others of your birth sex and age?*” on a 7-point Likert scale: 0 (more masculine), 3 (same), 6 (more feminine).

### Statistical analyses

ASR preference and behavior groups were compared on measures of age, ethnicity, right and left 2D:4D digit ratio, recalled childhood GNC and adulthood GNC (Analysis of Variance [ANOVA] for all continuous measures, and Kruskal–Wallis [H] for rank data [i.e., ethnicity and adulthood GNC]). Significant omnibus effects were followed up with Tukey HSD post hoc analyses (except Dwass-Steel-Critchlow-Fligner [DSCF] for rank data), and Cohen’s *d* was calculated as a measure of effect size for significant post hoc tests. Lastly, we used Pearson’s correlation to assess whether there was an association between right and left digit ratios on childhood GNC, and Spearman’s correlation to test for an association between right and left digit ratios with adulthood GNC. Alpha was set at *p* < .05.

## Results

### Age and ethnicity

We did not find any differences between ASR groups in age or ethnicity (See Table 1 for descriptive statistics). Specifically, age was not statistically different between either Anal Sex Role (ASR) preference groups, *F*(2, 124) = 2.6, *p* =.081, nor ASR behavior groups, *F*(2, 121) = 1.21, *p* = .303. Ethnicity also did not differ between ASR preference group, *H*(2) = 2.10, *p* = .35, nor for ASR behavior groups, *H*(2) = 3.74, *p* = .154.

**Table 1:** Descriptive statistics by ASR preference and behavior groups: mean (SD).

	Age (years)	Ethnicity	Right 2D:4D	Left 2D:4D	Childhood GNC	Adulthood GNC
Gay men (*n* = 125)	38.9 (13.9)	2.58 (3.39)	0.961 (0.054)	0.958 (0.052)	3.32 (0.41)	3.17 (1.61)
Anal sex role preference						
Bottom (*n* = 43)	35.6 (14.8)	3.49 (4.35)	0.976 (0.049)	0.964 (0.045)	3.24 (0.45)	3.7 (1.56)
Versatile (*n* = 51)	39.2 (13.3)	2 (2.59)	0.958 (0.057)	0.956 (0.051)	3.28 (0.39)	3.22 (1.64)
Top (*n* = 31)	43 (12.9)	2.32 (2.83)	0.945 (0.049)	0.951 (0.062)	3.49 (0.35)	2.35 (1.31)
Anal sex role behavior						
Bottom (*n* = 45)	37.2 (15.5)	3.41 (4.24)	0.966 (0.05)	0.953 (0.047)	3.24 (0.41)	3.8 (1.51)
Versatile (*n* = 33)	38.1 (13.8)	1.94 (3)	0.959 (0.058)	0.963 (0.053)	3.35 (0.4)	3.12 (1.67)
Top (*n* = 44)	41.4 (1.82)	2.2 (2.57)	0.956 (0.054)	0.958 (0.058)	3.38 (0.41)	2.5 (1.41)

Ethnicity was measured on a 13-point scale (1 = Caucasian, 2 = Black, 3 = Chinese, 4 = Filipino, 5 = Aboriginal, 6 = Latin American, 7 = Arab, 8 = Southeast Asian, 9 = West Asian, 10 = Korean, 11 = Japanese and 12 = other). Right- and left-hand digit ratios were calculated by dividing the length (mm) of the index finger (2D) by the length of the ring finger (4D). Childhood GNC was calculated based on an 18-item questionnaire, where *lower* scores indicate more gender nonconformity^10^. Adulthood GNC scores are derived from a on a 7-point Likert scale: 0 (more masculine), 3 (same), 6 (more feminine), thus *higher* scores indicate more gender nonconformity.

### ASR and digit ratios

**Figure 1:**
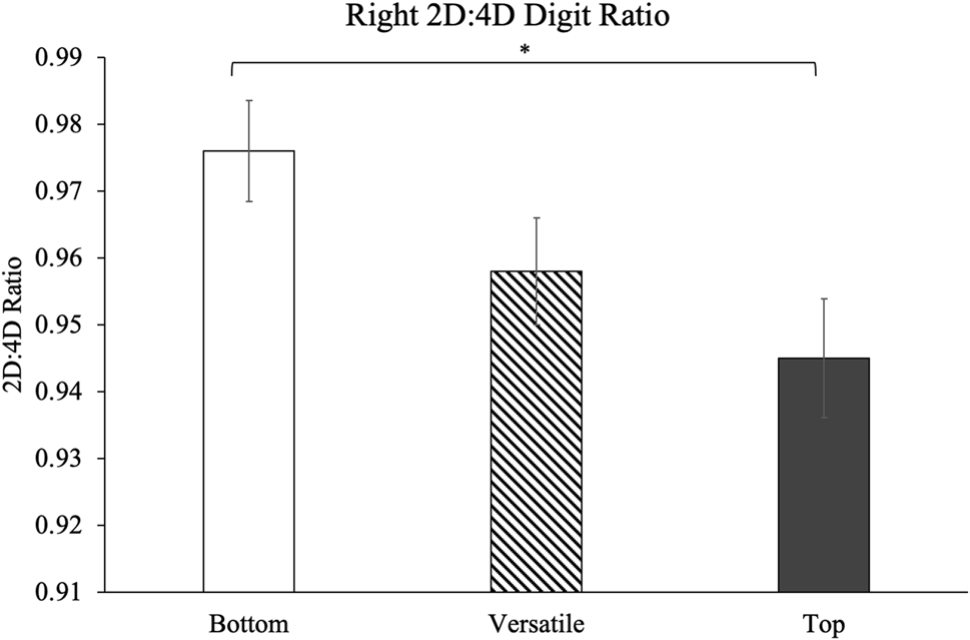
ASR preference groups differ in right digit ratios. ASR Bottoms have more female-typical (higher) right digit ratios than ASR Tops (*d* = 0.62); Versatiles were intermediate, such that they did not significantly differ from either ASR Tops or Bottoms. *Significant difference, *p* < .05.

A significant difference in right-hand digit ratios was found between ASR preference groups, *F*(2, 124) = 3.38, *p* = .037, such that ASR Tops displayed a lower (male-typical) ratio compared to Bottoms (Tukey *p* = .032, *d* = .63; See Fig. 1). ASR Versatiles were intermediate, such that they did not significantly differ from ASR Tops (Tukey *p* = .514) or Bottoms (Tukey *p* = .216). ASR preference groups did not differ in their left-hand digit ratios, *F*(2, 124) = 0.567, *p* = .57. ASR behavior groups did not differ in their right-hand, *F*(2, 121) = 0.412, *p* = .664, nor their left-hand digit ratios, *F*(2, 121) = 0.345, *p* = .709.

### ASR and recalled childhood GNC

**Figure 2:**
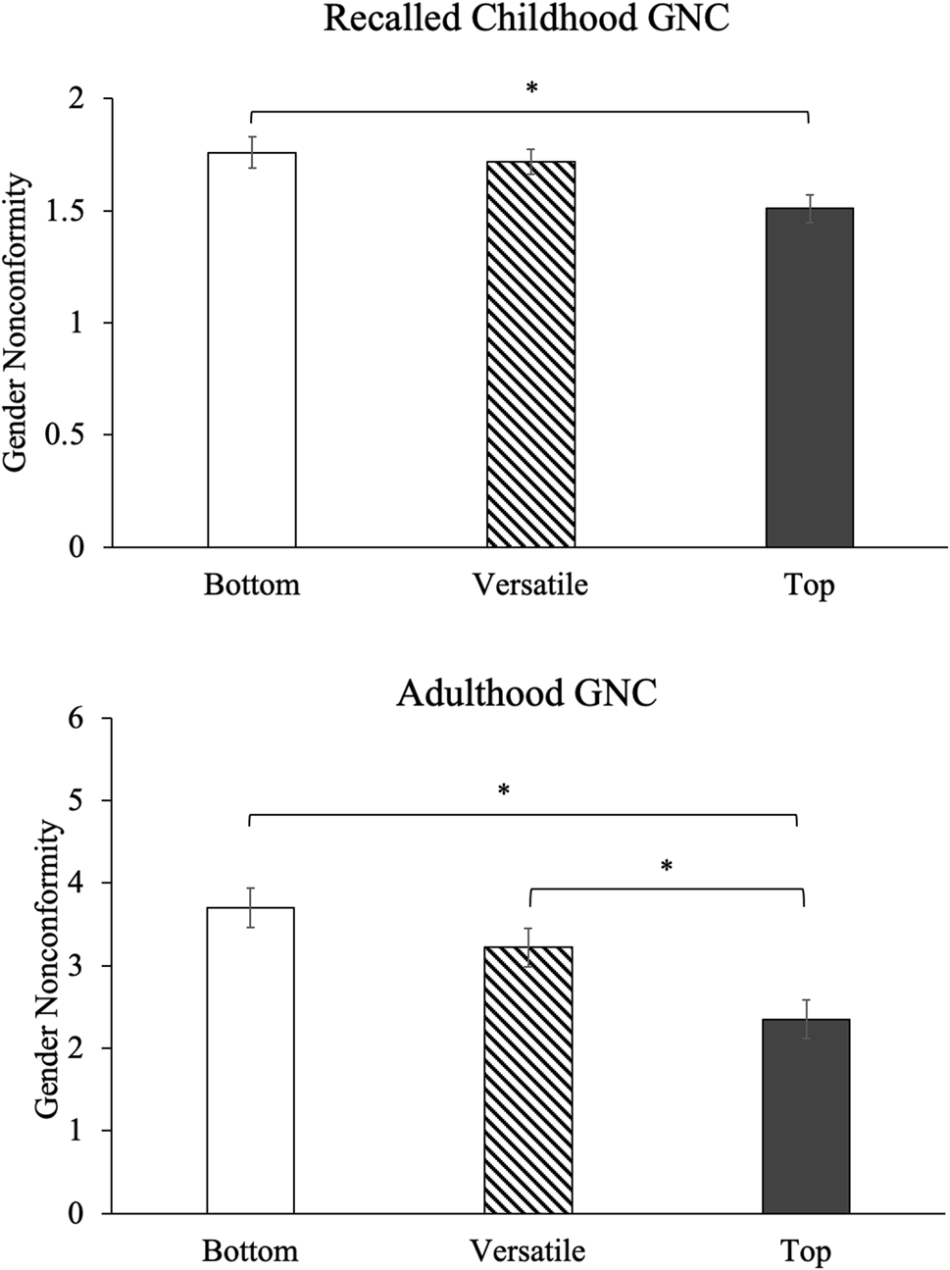
ASR preference groups differ in gender nonconformity. On both Recalled Childhood GNC and Adulthood GNC measures, ASR Tops rated themselves as more male-typical compared to Bottoms. On the Childhood GNC measure, Versatiles were intermediate, such that they did not significantly differ from Tops or Bottoms. On the measure of Adulthood GNC, Versatiles rated themselves as more GNC than Tops. For consistency across measures, score on the Recalled Childhood GNC scale were subtracted by 5 and multiplied by negative 1; Thus, a higher score on both the Childhood and Adulthood GNC indicates more gender *non*conformity. *Significant difference, *p* < .05.

ASR preference groups significantly differed in their Recalled Childhood Gender Nonconformity (GNC) scores, *F*(2, 124) = 4.47, *p* = .015, such that ASR Tops reported significantly higher gender conformity than ASR Bottoms (Tukey *p* = .027, *d* = 0.62; See Fig. 2). ASR Versatiles were intermediate, such that they did not significantly differ in Childhood GNC scores from either ASR Bottoms (Tukey *p* = .879), or Tops (Tukey *p* = .065). In contrast, ASR *behavior* groups did not significantly differ in Recalled Childhood GNC scores, *F*(2, 121) = 1.38, *p* = .258.

### ASR and adulthood GNC

On the measure of Adulthood GNC, ASR preference groups significantly differed, *H*(2) = 13.22, *p* = .001, such that Tops rated themselves as more masculine than both ASR Bottoms (DSCF *p* < .001, *d* = 0.94) and Versatiles (DSCF *p* = .013; See Fig. 2). Similarly, ASR behavior groups differed, *H*(2) = 14.89, *p* < .001; ASR Tops rated themselves more masculine than ASR Bottoms (DSCF *p* < .001), but did not significantly differ from ASR Versatiles (DSCF *p* = .098).

### Digit ratios correlate with GNC

**Figure 3:**
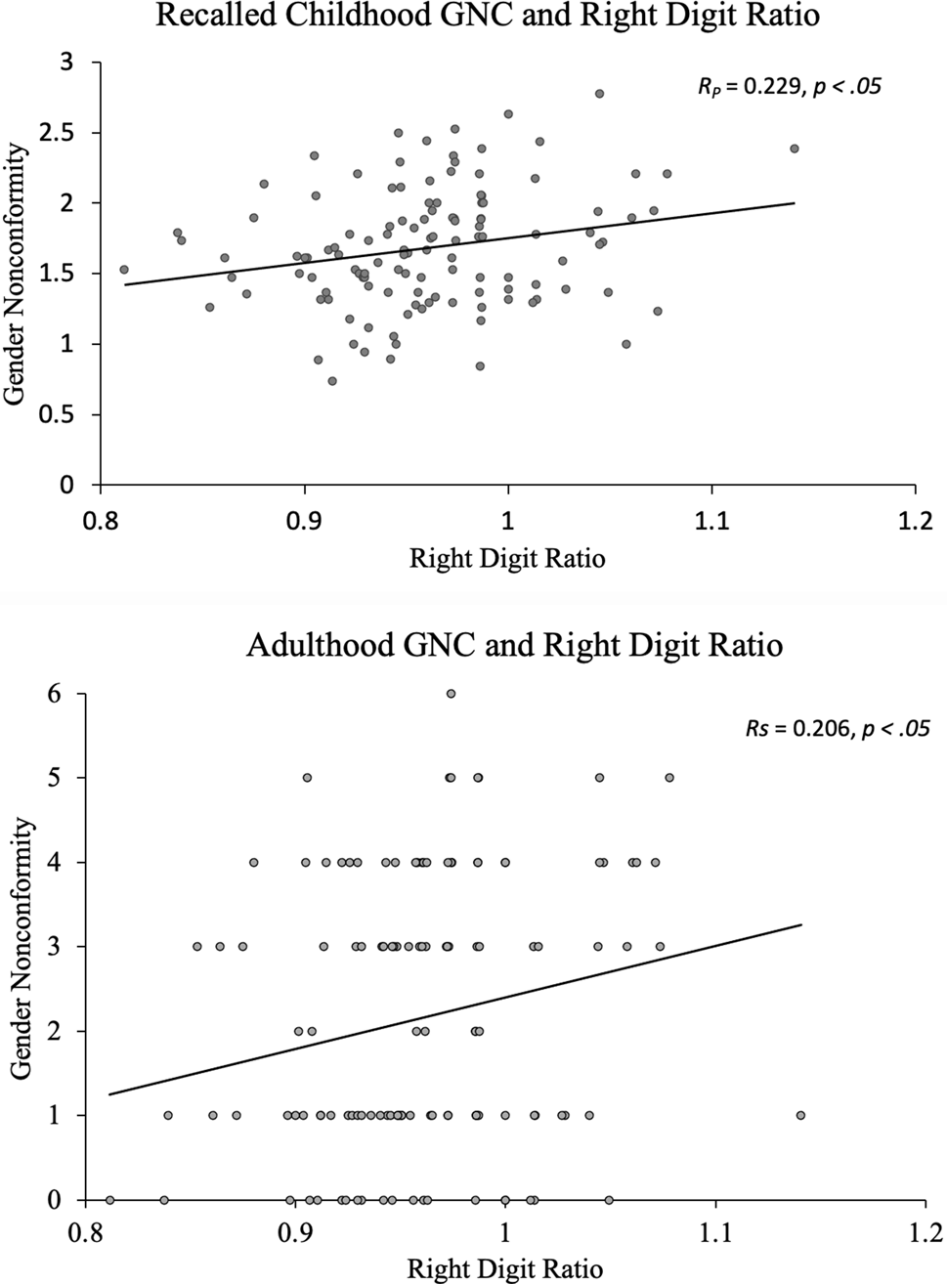
Right digit ratios correlate with gender nonconformity scores. A correlation between right digit ratios and Childhood GNC indicates that a more female-typical digit ratio is associated with higher gender nonconformity scores. Similarly, a positive correlation between right digit ratios and Adulthood GNC indicates that a more male-typical digit ratio is associated with higher gender conformity. For consistency across measures, score on the Recalled Childhood GNC scale were subtracted by 5 and multiplied by negative 1 for graphical depiction of results; Thus, a higher score on both the Childhood and Adulthood GNC graphs indicates more gender nonconformity.

Among the entire sample of gay men, right-hand digit ratios significantly correlated with both Recalled Childhood GNC (Pearson’s *R* =  − 0.229, *p* = .011) and Adulthood GNC, (Spearman’s *R* = 0.206, *p* = .021), such that lower (more male-typical) digit ratios were related to more gender conformity on both measures (see Fig. 3).

### Non-exclusive same-sex attraction, GNC and digit ratios

The principal analyses above assessed ASR differences in GNC and digit ratios among gay men, whereas other non-heterosexual men were excluded from these analyses as we hypothesized that there may be more variability in developmental processes influencing sexual orientation of men who display variation in same and opposite sex attraction and/or behavior. Here, we report analyses on the focal measures including all non-heterosexual men: self-identified gay men (*n* = 149), self-identified bisexual men (*n* = 24), and men who self-identified as “other” (*n* = 18; self-labels included: pansexual, asexual, nonbinary, genderqueer, queer, heteroflexible, questioning, trans, and various forms of “I don’t label myself”). Note that sample sizes (df) vary between measures due to participants declining to answer some questions.

### Gender nonconformity

On the Recalled Childhood GNC scale, the three groups of non-heterosexual men significantly differed, *F*(2, 184) = 13.5, *p* < .001; Post hoc Tukey tests indicated that all groups differed from one another (*ps* < .05), with bisexual men reporting the lowest childhood GNC followed by gay men, and the “other” group reported the highest GNC.

Group differences were also found on the adulthood GNC measure, *F*(2, 192) = 10.7, *p* < .001, and post hoc tests indicated that while bisexual and gay men did not significantly differ (*p* = .166), both of these groups report lower GNC than the “other” group of non-heterosexual men (*ps* < .001).

### Digit ratios

The three groups of non-heterosexual men did not differ in right, *F*(2, 185) = 0.44, *p* = .643, or left, *F*(2, 185) = 0.04, *p* = .959, digit ratios.

### Digit ratio and GNC correlations

Right-hand, but not left-hand, digit ratios significantly correlated with recalled childhood GNC, *r* = − 0.191, *p* = .01), indicating that higher recalled GNC is associated with more female-typical digit ratios. The correlation between right-hand digit ratio and adulthood GNC was trending, *r* = 0.141, *p* = .054.

### ASR and digit ratios

With all non-heterosexual men included in the analyses (i.e., those who also reported on ASR and digit ratios), no ASR preference group differences were found in right, *F*(2, 169) = 2.06, *p* = .13, or left, *F*(2, 169) = 0.63, *p* = .54, hand digit ratios. Similarly, ASR behavior groups did not significantly differ in right, *F*(2, 159) = 0.12, *p* = .89, or left, *F*(2, 169) = 0.37, *p* = .69, digit ratios when all non-heterosexual men were included in the analyses.

## Discussion

We replicated the finding that ASR groups differ in both recalled childhood GNC and adulthood GNC, as gay men with an insertive ASR preference (self-identified Tops) were more gender conforming than men with a receptive ASR preference (self-identified Bottoms). Digit ratios on the right-hand differed among ASR preference groups, such that Tops had lower (male-typical) digit ratios than Bottoms, while self-identified Versatiles were intermediate, such that they did not significantly differ from either group. Finally, we found that across the entire sample of gay men, 2D:4D ratios correlated with both recalled childhood and adulthood measures of GNC, such that a more male-typical (lower) 2D:4D was associated with more gender conformity. Altogether, these findings support a role for prenatal androgens in influencing ASR preference and gender conformity of gay men.

The finding that 2D:4D ratios differ in a subset of gay men based on ASR and GNC corresponds well to the literature on 2D:4D ratios in sexual orientation in women: self-identified “femme” and “butch” lesbians differ in their average digit ratios such that the more gender conforming group (“femmes”) had more female-typical (higher) 2D:4D ratios^3^. The present and previous research together suggest that prenatal androgens predispose humans to be attracted to females in adulthood, that this effect is sufficient to detect differences in 2D:4D between lesbians and straight women, and explains why the vast majority of men are heterosexual. If all males are exposed to sufficient androgens to maximize this effect, then some other factor(s) result in same-sex attraction among a minority of men. However, even within gay men, prenatal androgens may still have a modulatory effect on gender conformity and ASR preference. We take it as a given that variation in prenatal androgens cannot account for all variation in human sexual orientation, which is surely subject to many converging influences, including genes, prenatal factors in addition to androgens and, to some extent, cultural tolerance for same-sex preferences. This hypothesis fits well with recent literature pointing towards multiple distinct developmental pathways underlying same-sex sexual orientation in men^11^.

We also found correlations between right-hand 2D:4D and GNC measures, such that higher digit ratios (indicating less prenatal androgen exposure) are associated with increased recalled childhood and adulthood GNC. These findings are consistent with the literature demonstrating a relationship between digit ratios and gender among populations with gender dysphoria as well as in the general population^12–17^. Indeed, a recent large scale internet study (*n* > 200,000) indicated males who reported that they “felt female” had higher digit ratios (more female-typical) than those who “felt male”^18^. Altogether, this research implicates a contributory role for the prenatal endocrine environment in the development of gender traits and gender identity. Alternatively (although not mutually exclusively), prenatal androgens may mediate a third variable such as sexual orientation and/or body type which may influence gender conformity.

The present findings are consistent with previous literature showing that gay men with an insertive ASR preference display other indicators of high androgen exposure, including an earlier pubertal onset, increased height and body hair compared to both heterosexual men and other gay men^19^. These findings together with the current study suggest that gay men with an insertive ASR may be exposed to higher levels of androgens throughout their lifespan, rather than, or in addition to, receptive ASR gay men being exposed to lower androgens prenatally. As previously proposed^20,21^, it is possible that both high and low prenatal androgen exposure could alter androphilia as an inverted-U relationship has been found between androgens and androphilia among other mammalian species.

Only the right-hand, not the left-hand, 2D:4D measurements differed between ASR groups, and it was specifically ASR preference, not behavior, groups who differed in their digit ratios. The finding that the right but not the left-hand show group differences in 2D:4D corresponds well with previous research: the sex difference in 2D:4D is more consistently present in the right than the left-hand, and similarly, groups who differ in prenatal androgen levels or sensitivity also show differences in digit ratios more consistently in the right-hand (e.g.,^22^; reviewed in^3^). While the reasons for this laterality remain unknown, this is not the first difference in lateralization reported in the study of sexual differentiation. Indeed, there are other known lateralization differences between the sexes (e.g., handedness in humans, medial amygdala volume in rodents)^23–25^. Consistent with the present study, previous research has also indicated ASR preference, but not behavior, group differences on other markers linked to sexual orientation (e.g., fraternal birth order effect^7^). While there is generally high concordance between preference and behavior, is likely that preference/fantasies correspond more to internal factors, whereas behavior is more constrained by external considerations, such as the preferences of sexual partners, and/or socio-cultural factors associated with masculinity and sex role^26^.

The primary analyses in the present study evaluated ASR group differences in GNC and digit ratios among gay men, whereas other non-heterosexual men were excluded from these analyses. This approach was taken due to the hypothesized increase in heterogeneity in the development of sexual orientations among these groups that are more variable in same and opposite sex attraction and/or behavior. Our analyses of gay, bisexual and other non-heterosexual self-identities (i.e., pansexual, heteroflexible, queer, etc.) suggest more variability in these groups. For instance, these three groups of non-heterosexual men differ in GNC: both self-identified bisexual and gay men report lower recalled childhood and adulthood GNC compared to the “other” group of non-heterosexual men, and bisexual men report lower childhood GNC compared to gay men. Although these three groups do not show any differences in digit ratios, ASR group differences do not reach significance when all non-heterosexual men are included in the analyses. GNC measures continued to correlate with right-hand digit ratios among the sample of all non-heterosexual men. These results are consistent with other correlates of same-sex attraction; exclusive vs non-exclusive same-sex oriented men differ on various physiological and behavioral measures^27,28^. Thus, while it is possible that prenatal androgens contribute to the sexual orientation of some non-exclusive same-sex oriented men, this prior work together with the present study suggests that the development of same-sex attraction among bisexual and other non-heterosexual men likely differs and/or may be more variable than gay men.

It is important to note the limitations of the present study that should be taken into consideration. Firstly, this study is a cross sectional study that relied on a convenience sample. The sample is also largely homogeneous with respect to ethnicity (~ 80% White), and prior work has indicated that there are ethnic differences in digit ratios (e.g.,^5^), likely reflecting the role of genetics on digit ratios. There is also debate surrounding the validity of digit ratios as a retrospective marker of prenatal androgen, as some argue against the use of digit ratios and/or the validity of the measure as a marker of prenatal androgens^29^. We recently reviewed the evidence supporting the association between digit ratios and prenatal androgens^3^, and argue that the imperfection of the measure does not nullify its usefulness. Instead, given the few alternatives in studying the human prenatal endocrine environment, it would be more prudent to refine the technique to improve consistency and studying other possible influences on the development of this measure (i.e., genetics) to increase the usefulness and validity of the marker. There have also been concerns surrounding the use of indirect measures of digit ratios compared with direct measures^30,31^. We contend that photocopies with standardized instructions and ruler, as in the present study, offers advantages over direct measures with calipers because photocopies allow replication of the basic measure, and they avoid any bias an experimenter measuring digits directly from the participant might acquire during their interaction. We, as well as other labs, have used this measure in prior publications, showing consistent sex differences and sexual orientation differences among females^3^.

In conclusion, we found that a retrospective approximation of prenatal androgens, the 2D:4D ratio, differs among ASR preference groups, suggesting that gay men who prefer a receptive ASR and who tend to be more gender nonconforming were exposed to a lower level of prenatal androgen than gay men who prefer an insertive ASR, who tend to be more gender conforming. These results support the neuroendocrine hypothesis in that at least a *subset* of gay men may owe their sexual orientation to a reduction in prenatal androgens, although it is possible that high levels of prenatal androgens could also increase odds of androphilia among men. Finally, these results are also consistent with the hypothesis that there are multiple developmental pathways underlying male sexual orientation, and that future research should not consider gay men as a homogenous group.
